# Survival Prediction in Brain Metastasis Patients Treated with Stereotactic Radiosurgery: A Hybrid Machine Learning Approach

**DOI:** 10.3390/brainsci15030266

**Published:** 2025-03-01

**Authors:** Tuğçe Öznacar, İpek Pınar Aral, Hatice Yağmur Zengin, Yılmaz Tezcan

**Affiliations:** 1 Department of Biostatistics, Faculty of Medicine, Ankara Medipol University, 06570 Ankara, Turkey; 2 Radiation Oncology Clinic, Faculty of Medicine, Ankara Yıldırım Beyazıt University, 06800 Ankara, Turkey; ipekpinararal@aybu.edu.tr (İ.P.A.); yilmaztezcan@aybu.edu.tr (Y.T.); 3 Department of Biostatistics, Faculty of Medicine, Hacettepe University, 06230 Ankara, Turkey; yagmurzengin@hacettepe.edu.tr

**Keywords:** brain metastasis, survival prediction, machine learning, hybrid model, meta model

## Abstract

Objectives: Accurate survival prediction for brain metastasis patients undergoing stereotactic radiotherapy (SRT) is crucial for personalized treatment planning and improving patient outcomes. This study aimed to develop a machine learning model to estimate survival times, providing clinicians with a reliable tool for making informed decisions based on individual patient characteristics. The goal was to compare the performance of multiple algorithms and identify the most effective model for clinical use. Methods: We applied a hybrid machine learning approach to predict survival in brain metastasis patients treated with SRT, utilizing real-world data. Four algorithms—XGBoost, CatBoost, Random Forest, and Gradient Boosting—were compared within a meta-model framework to identify the most accurate for survival prediction. Model performance was evaluated using metrics such as MSE, MAE, MAPE, and C index. Results: XGBoost outperformed all other algorithms, achieving an MSE of 0.14%, MAE of 0.10%, and MAPE of 0.093%, with a high C-index of 100%. CatBoost showed reasonable performance, while Gradient Boosting had higher error rates (MSE of 6.99%, MAE of 21.04%, MAPE of 19.29%). Random Forest performed the weakest, with the highest MSE (14.39%), MAE (30.23%), and MAPE (33.58%). Conclusion: Inputting relevant clinical variables into the model enables clinicians to obtain highly accurate survival predictions for patients with brain metastasis. This enhances clinical decision making by providing a more precise understanding of expected outcomes. The XGBoost-based hybrid model showed exceptional accuracy in predicting survival for brain metastasis patients after SRT, offering valuable support for clinical decision making. Integrating machine learning into clinical practice can improve treatment planning and personalize care for these patients.

## 1. Introduction

The risk of developing brain metastases following a cancer diagnosis ranges between 30 and 40% [1]. The main treatment approaches include surgery, whole-brain radiotherapy (WBRT), or stereotactic radiotherapy (SRT). Key factors influencing patients’ treatment preferences include life expectancy, the number and total volume of metastases, control of the primary tumor, comorbid conditions, anticipated treatment toxicities, and individual patient preferences [2,3]. The treatment processes for brain metastases, which have a direct impact on patients’ quality of life and survival, continue to evolve with the introduction of novel therapeutic strategies.

Survival expectations play a critical role in determining treatment strategies for brain metastases. However, algorithms capable of accurately predicting survival remain in the development phase [4]. This study aims to test an algorithm designed to estimate the expected survival times of patients with brain metastases following SRT.

In recent times, artificial intelligence (AI) and machine learning have had great impact in the field of medicine by improving quality of clinic decision-making processes. Particular machine learning algorithms in medicine could be the tools of the future, as there is a promise of understanding sophisticated database patterns for the improved diagnosis, treatment, and prognosis of individual patients. The role of AI in medicine enables one to better address the management of complex diseases such as cancer by assisting physicians in treatment planning and management. In all circumstances, these technologies could lead to increased life quality and better results in treatment [5,6,7,8,9,10].

In this context, our study created a meta-model and a hybrid model that employs four machine learning algorithms, which are XGBoost, CatBoost, Random Forest, and Gradient Boosting—were compared within a meta-model framework to identify the most accurate for survival prediction using Python version 3.10.12. The main goal of this study was to develop a model that can estimate the survival time of a patient with brain metastases who has undergone stereotactic radiotherapy and to validate its accuracy. In addition, this study sought to enhance the success of the hybrid model by determining the effect of different algorithms on the meta-model. This research is aimed at supporting the introduction of AI-based approaches in medicine practices as clinical decision-supporting systems.

## 2. Materials and Methods

This retrospective study analyzed data from 109 patients who underwent stereotactic radiotherapy (SRT) for brain metastases at the Radiation Oncology Department of Ankara Bilkent City Hospital between 1 January 2019, and 1 September 2022. Given the retrospective nature of this study, all eligible patients treated within this period were included. After obtaining ethical approval from the Ethics Committee of Ankara Bilkent City Hospital (Ethics Approval No. E1-22-2874, dated 7 September 2022), data were collected from patient interview records, electronic hospital records, radiotherapy details, dose–volume histograms, and other relevant clinical parameters. Information regarding patients’ demographic characteristics, radiological and pathological findings, radiotherapy parameters, recurrence status, and survival outcomes was meticulously recorded. The data collection and analysis were conducted by the responsible and assisting researchers, and all data were stored in SPSS v.26 in the archives of the Radiation Oncology Clinic at Ankara Bilkent City Hospital. The study does not involve any potential confounders. This study was conducted in accordance with the principles outlined in the Declaration of Helsinki.

### 2.1. Patient Follow-Up and Endpoints

The primary endpoint of the study was the prediction of overall survival (OS) using the developed algorithm. OS was defined as the time from the end of stereotactic body radiotherapy (SBRT) to the death of the patient. The end date of SBRT was considered the starting point for OS. For patients who were still alive, the endpoint was recorded as the date of their last follow-up, while for deceased patients, the endpoint was the recorded date of death.

### 2.2. Stereotactic Radiotherapy Technique

Patients were treated using the Eclipse treatment planning system (Varian Oncology System, Inc., Palo Alto, CA, USA). Prior to SRT, all patients underwent high-resolution brain MRI scans, and target volumes were delineated by fusing the MRI images with the treatment planning CT. The gross tumor volume was defined, and a margin of 1–2 mm was applied to determine the planning target volume. A stereotactic radiotherapy dose of 1–35 Gy was delivered, depending on the clinical parameters of each patient.

Patients’ gender was recorded as a potential factor influencing survival. The presence of extracranial metastases was documented for all patients. Immunotherapy administration concurrent with stereotactic radiotherapy was noted. The number of brain metastases was counted and included in the analysis. The gross tumor volume and its derived total tumor volume were calculated. The conformality index (CI) and the gradient index (GI) were computed for each treatment plan. Additionally, tumor recurrence was assessed during follow-up as a critical clinical outcome.

### 2.3. Hybrid Model Approach

Four machine learning algorithms were used in this research with the aim of estimating how long patients who had already developed brain metastasis were likely to live. Hyperparameter optimization was performed for each of these models, and the stacking method was used to aggregate the results and build a meta-hybrid model for prediction. The detailed steps taken in this process are explained below.

The dataset has been transferred to the Python environment for analysis. Firstly, independent variables were divided into categorical and numerical types. For categorical variables, the gender of the patients, status of the primary disease, existence of extra cranial metastases, presence of any immunotherapies, and recurrence of the disease were included, whereas for numerical variables, measurements related to the size, volume, and total doses of tumors as well as other biomarkers to the administration and progression of the disease were included.

The Iterative Imputer technique played a role in completing the missing data in the dataset. This technique predicts the values of other variables so as to fill in the gaps of missing data [11].

The survival time was logarithmically transformed to make an appropriate adjustment considering the distribution of the OS [12]. In order to determine the validity of the model, the K-Fold Cross-Validation method was employed [13]. This technique takes 5 equal parts of the dataset, and each part acts as the training or test dataset at different times. This validity method describes a process where the model is developed in every repetition and the outcome is compared with the test set, and it improves the model’s generalization capability and reduces the chances of it overfitting. Hyperparameters were optimized using GridSearch.

- XGBoost Regressor: A gradient boosting algorithm that is mostly preferred to obtain high-quality estimates [14]. During research, the hyperparameters (n_estimators = 100), learning rate (learning_rate = 0.05), and the maximum tree depth (max_depth = 7) were optimized.
- Gradient Boosting Regressor: Implementation of the same idea was used, but instead to create smaller trees [15]. With regard to adequate predictive outcome, the optimal parameters were specified as n_estimators = 100, learning_rate = 0.1, and max_depth = 4.
- Random Forest Regressor: This is a combination of several decision trees in the model [16]. The optimized hyperparameters for this model included the number of trees to be used (n_estimators = 100), the highest depth of trees (max_depth = 4), and the minimum number of samples needed to split a node (min_samples_split = 5).
- CatBoost Regressor: This is an algorithm particularly good with categorical datasets [17]. After hyperparameter optimization, we obtained the following parameters: depth = 5, learning_rate = 0.05, and iterations = 100 for effective and accurate predictions.

### 2.4. Stacking Meta-Model

The predictions obtained from different individual models were utilized as inputs for the meta-model. This meta-model was trained to enhance its predictions by merging the outputs of these models. During the construction of the hybrid model, each algorithm was implemented separately, allowing the meta-model to capture the strengths of distinct individual models. Functioning as the final predictor, the meta-model combines the predictions from the individual models and generates a unified final output. This technique has demonstrated its effectiveness in improving prediction accuracy by efficiently leveraging the unique capabilities of the individual models.

### 2.5. Evaluation Metrics

A variety of performance metrics were used to assess the final model including Mean Squared Error (MSE), Root Mean Squared Error (RMSE), Mean Absolute Percentage Error (MAPE), R-squared (R²), Explained Variance Score, and Concordance Index (C-index) [18,19,20,21,22,23].

### 2.6. Visualization and Analysis of Model Performance and Feature Relationships

To evaluate the relationship between variables, the performance of our model, and the significance of the variables used in the model, we employed various statistical and visualization techniques including evaluating the correlation matrix, visualization of the relationships with the scatter plots, and using SHAP analysis to evaluate the feature importance. In particular, it was important to measure the significance of features in the model. Therefore, we used the SHAP (SHapley Additive exPlanations) approach, which offers a comprehensive method to understanding how individual features contribute to the predictions of the model and how they interact with each other, providing both global and local interpretability [24,25,26].

All analyses were conducted using Python version 3.10.12.

**Figure 1:**
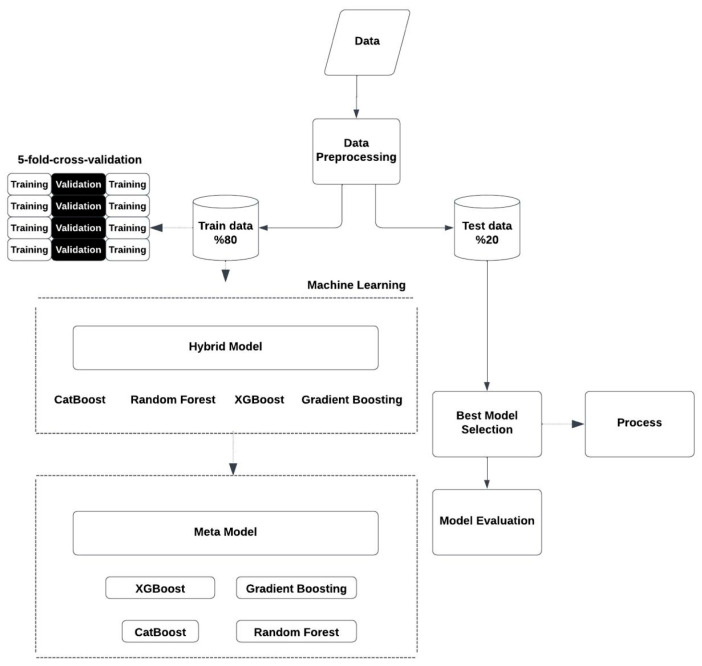
Flow chart.

The entire process is shown in Figure 1.

## 3. Results

The performance of the proposed hybrid model was evaluated using multiple regression metrics, as summarized in Table 1. Among the four meta-model configurations, XGBoost demonstrated the best performance, achieving near-perfect predictive accuracy. Its MSE was exceptionally low at 0.14%, while the MAE was 0.10%, and the MAPE was only 0.093%. The model also achieved a perfect C-index of 100%, indicating superior consistency and stability. These results highlight XGBoost’s dominance in both predictive accuracy and robustness.

CatBoost, while slightly less effective, delivered reasonable performance. Gradient Boosting showed moderate predictive capability, with an MSE of 6.99%, RMSE of 26.43%, MAE of 21.04%, and a MAPE of 19.29%. Although its R² value of 91.88% and C-index of 94.05% suggest a decent fit, its higher error rates reduce its overall reliability.

The Random Forest meta-model performed the poorest, with the highest error rates: an MSE of 14.39%, RMSE of 37.94%, MAE of 30.23%, and an MAPE of 33.58%. These metrics underscore its limited predictive accuracy in this context.

**Figure 2:**
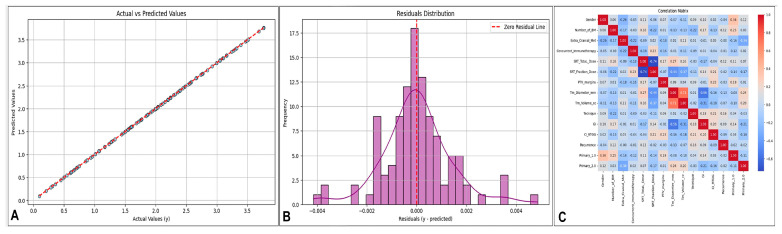
(**A**) Scatter plot. (**B**) Residuals distribution. (**C**) Heat map.

Figure 2 provides a comprehensive evaluation of the model’s performance through diagnostic graphics and correlation analysis. The scatterplot comparing actual and predicted values demonstrates high prediction accuracy, with most points closely aligned along the 45-degree line, indicating strong model performance. Residual analysis reveals that errors are normally distributed around zero, with no significant skewness or heteroscedasticity, confirming the model’s stability. The correlation heatmap highlights the relationships among features, offering valuable insights into feature dependencies and their potential impact on the model’s performance.

**Figure 3:**
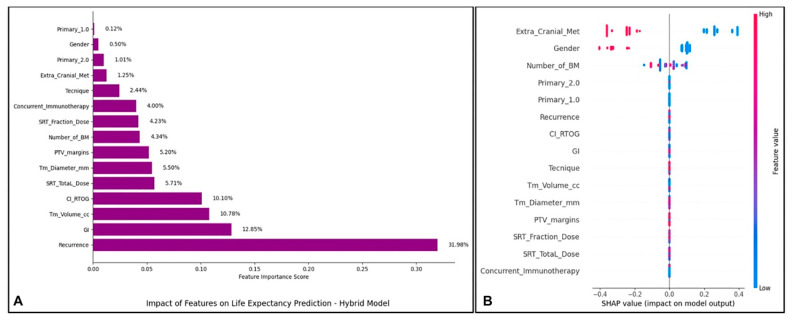
(**A**) Feature importance plot for the meta-model XGBoost. (**B**) SHAP analysis for the meta-model XGBoost.

Figure 3 illustrates the role of individual features in predicting survival time using the hybrid XGBoost meta-model. Panel A presents relative feature importance scores, while Panel B showcases a SHAP summary plot detailing the direction and magnitude of feature effects on the prediction model.

Panel A highlights feature importance, with Recurrence contributing the most (32%), followed by GI (12.85%) and CI_RTOG (10.10%). Features such as Tm_Volume_cc and Concurrent_Immunotherapy show moderate influence, whereas Primary_1.0 and Gender have minimal impact.

Panel B provides a detailed SHAP analysis, indicating that higher values of Recurrence, Tm_Volume_cc, and Concurrent_Immunotherapy are associated with increased risk (shorter survival), while lower values reduce risk. Other features, like CI_RTOG and SRT_Total_Dose, show variable effects depending on their range.

## 4. Discussion

This study evaluated the significance of a hybrid machine learning model for predicting survival outcomes in patients with brain metastases undergoing SBRT. The results highlight the value of employing multiple machine learning algorithms to enhance prediction accuracy and the practicality of these models in clinical settings. Specifically, tumor recurrence emerged as the most significant predictor of survival, reflecting a more aggressive disease biology and resistance to treatment. Tumor volume was identified as the second most influential factor, underscoring the challenges of treating larger tumors with stereotactic radiotherapy due to their inherent biological aggressiveness. Furthermore, dosimetric indices used in radiotherapy planning, such as GI and CI, played a crucial role in optimizing treatment outcomes. These findings are consistent with the existing literature and emphasize the importance of developing predictive models that assist in clinical decision making [27,28,29,30,31,32,33].

This study evaluated the significance of a hybrid machine learning model for predicting survival outcomes in patients with brain metastases undergoing SBRT. It highlights the value of employing multiple machine learning algorithms to enhance prediction accuracy and the practicality of these models in clinical settings.

CatBoost, another tree-based gradient boosting model, showed strong predictive performance, though it was slightly less effective than XGBoost.

The performances of Gradient Boosting and Random Forest, while still significant, were relatively lower in this study. This is consistent with the work of Chen et al. (2024), who noted that ensemble methods like XGBoost are more effective due to their ability to reduce overfitting and optimize feature usage [34].

However, there are some limitations to our work. The relatively small sample size of 109 patients, recruited from a single site, raises questions about the generalizability of the results. Nevertheless, it should be noted that the dataset used in this study represents real-life data, which inherently limits sample size. Larger, multi-center datasets will be necessary to validate and expand the findings. Furthermore, the retrospective design introduces potential biases due to the heterogeneity of clinical parameters, a concern also raised in similar studies, such as Zhou et al. (2024), who emphasized the need for prospective validation of machine learning models in cancer treatment [35].

This study contributes to the growing body of literature on the application of machine learning in healthcare, particularly in predicting patient outcomes. Although a lot of previous research has focused on single algorithms for survival predictions, our hybrid approach suggests that relying on a single model may lead to biased results. This is supported by Zhu et al. (2024), who found that hybrid models offer better accuracy and reproducibility in predicting breast cancer recurrence [36]. Similarly, Minardi et al. (2025) employed machine learning techniques like LASSO to predict outcomes in patients with ruptured anterior communicating artery aneurysms. Their study analyzes a combination of preoperative and intraoperative factors to predict postoperative patient outcomes and emphasizes the significance of factors such as preoperative scores and intraoperative conditions. Both studies underscore the crucial role of machine learning in personalized patient care and its potential to improve clinical decision making and outcomes [37].

Compared with other works, such as the study by Fang et al., which used a large cohort of 20,249 patients to predict lung metastasis risk in esophageal carcinoma patients, our study focuses on the survival prediction of brain metastasis patients using a smaller sample size. While Fang’s work demonstrates the generalization capability of machine learning with robust metrics (AUC = 0.803, accuracy = 0.849), our model based on XGBoost showed a high C-index of 1, emphasizing its clinical accuracy. Fang’s research highlights large-scale generalization, whereas our work focuses on individualized prediction and treatment planning [38].

The work of Munai et al. developed a machine learning model for predicting brain metastasis risk in small-cell lung cancer patients, achieving notable results with the Random Forest model (AUROC = 0.896, AUPRC = 0.900) [39]. While their model focuses on risk estimation for developing brain metastasis, our study aims to predict survival outcomes for patients already diagnosed with brain metastasis, using a hybrid model to enhance prediction accuracy.

Additionally, Habibi et al. (2024) explored the prediction of treatment response and local control failure in brain metastasis patients treated with frameless stereotactic radiosurgery [40]. While their focus was on treatment response prediction, our study takes a broader approach, developing a hybrid model to estimate overall survival in patients with brain metastases, specifically considering those who have undergone SBRT.

Yichu et al. conducted a study on the use of radiomics and machine learning in predicting brain metastasis development in lung cancer patients [41]. Their work demonstrated the value of intratumoral and peritumoral features for prediction. In contrast, our study combines both clinical and radiomic variables to predict survival in patients with brain metastasis from various cancers, expanding the applicability of the model.

Research by Rappaport et al. (2024) utilized a semi-automatic machine learning approach to measure metastatic burden in preclinical models, offering useful insights into metastatic disease progression [42]. Unlike their preclinical focus, our study aims to translate AI-based prediction methods into clinical practice, improving treatment planning and patient management.

Finally, Liu et al. (2024) used a machine learning model combining radiomic and clinicoradiological features to predict perineural invasion in intrahepatic cholangiocarcinoma, showcasing the prognostic value of AI [43]. Similarly to Liu et al., our study demonstrates the potential of AI in tailoring treatments to individual patients, particularly in the context of brain metastases. Both studies underline the increasing relevance of AI for precision medicine in cancer care.

This increasing body of literature, including studies like those of Radhakrishnan et al. (2024), and Sulaiman et al. (2024), shows the promise of hybrid models in improving diagnostic accuracy across various domains [44,45]. While our study contributes to advancing hybrid machine learning techniques, it also opens avenues for future research aimed at improving model performance and clinical applicability.

Izonin et al. developed an ensemble machine learning model to improve the accuracy of biomedical data analysis, focusing on heart rate prediction for stress level assessment. Their model, based on Support Vector Regression (SVR) and cascading principles, achieved over 20 times higher accuracy compared to existing methods, with a significant reduction in training time. In contrast, our study focused on predicting survival outcomes for brain metastasis patients undergoing SRT using a hybrid machine learning approach [46].

One of the primary limitations of this study is the relatively small sample size, which is largely due to the inherent challenges of recruiting patients with brain metastases. Given the retrospective nature of the study, data collection was restricted to the available patient population within the specified timeframe. Future research should aim to include a larger patient cohort to improve the model’s performance in terms of accuracy and generalizability. A multicenter approach could help address this limitation by incorporating data from different institutions, thereby increasing the sample size and enhancing the model’s robustness across diverse patient populations. Additionally, as more patient data become available over time, continuous model training and validation could further refine predictive accuracy and improve clinical applicability. Another avenue for future studies is the integration of additional clinical and biological variables, which may enhance the model’s predictive capabilities and provide deeper insights into patient outcomes. These improvements would contribute to the development of more precise and individualized treatment strategies for patients with brain metastases.

## 5. Conclusions

All other individual machine learning algorithms showed comparatively lower performance than the proposed hybrid model for all the metrics evaluated, which shows high predictive capabilities. The low MSE of 0.0000019 and RMSE of 0.0014 for the hybrid model reflects its superiority over the other methods in terms of accuracy. Furthermore, the perfect C-index = 1.0 further supports its ability to predict outcome rankings, indicating that the model is effective and reliable. The near-zero error values, coupled with an R² of 99.9%, highlight the hybrid model’s capacity to explain and predict survival with high confidence. When a patient presents with brain metastasis, the model, developed based on the relevant clinical variables identified in the study, provides highly accurate survival predictions. This approach enables clinicians to estimate the patient’s survival time based on individual characteristics, thereby supporting clinical decision making with a more precise understanding of expected outcomes. By integrating machine learning models into clinical practice, physicians can enhance treatment planning, personalize care, and ultimately improve patient outcomes.

## Figures and Tables

**Table 1:** The performance of the proposed hybrid model was evaluated using multiple regression metrics.

Metric	XGBoost Meta-Model	CatBoost Meta-Model	Gradient Boosting Meta-Model	Random Forest Meta-Model
MSE	0.0000	0.0211	0.0699	0.1439
RMSE	0.0014	0.1451	0.2643	0.3794
MAE	0.0010	0.1133	0.2104	0.3023
MAPE	0.0931	10.3640	19.2890	33.5808
MedAE	0.0007	0.0866	0.1951	0.2526
R² Score	0.9998	0.9755	0.9188	0.8328
Explained Variance Score	0.9998	0.9755	0.9188	0.8328
C-index	1.0000	0.9684	0.9405	0.9212

## Data Availability

We will share our data upon request.

## Abbreviations

The following abbreviations are used in this manuscript:

SRT	Stereotactic Radiotherapy
OS	Overall Survival
SBRT	Stereotactic Body Radiotherapy
MSE	Mean Squared Error
RMSE	Root Mean Squared Error
MAPE	Mean Absolute Percentage Error
R²	R-squared
C-index	Concordance Index
SHAP	SHapley Additive exPlanations
GI	Gradient Index
CI	Conformity Index
BM	Brain Metastasis
